# From mechanism to phenotype: What fits in a basket trial

**DOI:** 10.1002/epi.70086

**Published:** 2026-01-05

**Authors:** Kette D. Valente

**Affiliations:** ^1^ Faculdade de Medicina da Universidade de São Paulo São Paulo Brazil; ^2^ Hospital das Clínicas da Faculdade de Medicina da Universidade de São Paulo São Paulo Brazil

**Keywords:** basket trial, epilepsy, master protocol, rare disorders

Developmental and epileptic encephalopathies (DEEs) are etiologically heterogeneous, pharmacoresistant, early life epilepsies and neurodevelopmental plateau, stagnation, or regression.^1^ Approximately 50% of affected individuals have identifiable genetic causes, with more than 1000 implicated genes.^2^

Conventional epilepsy trials have historically been anchored in three dominant paradigms: syndrome‐specific (e.g., Dravet syndrome^3^), etiology‐specific (e.g., tuberous sclerosis complex and mTOR inhibitors^4^), and seizure‐type‐specific designs (e.g., focal onset seizures^5^). Although scientifically rigorous, these paradigms exclude most individuals with rare DEEs whose phenotypes do not map neatly onto categorical trial structures, leaving treatment decisions reliant on off‐label or compassionate‐use therapies with limited supporting evidence.^6^

Master protocols—basket, umbrella, and platform trials—were originally developed in oncology to overcome analogous constraints enabling multiquestion trials under one infrastructure.^7^ Umbrella trials evaluate multiple targeted therapies within a single disease defined by shared clinical context; platform trials allow continuous addition or discontinuation of arms using adaptive rules (Figure 1). Their innovative designs provide a framework to overcome methodological and regulatory barriers that constrain evidence generation for rare disorders. They tailor interventions to patient‐specific factors, including genetic variants, biomarkers, and other treatment‐relevant characteristics.^7^ Monogenic epilepsies with mechanistic convergence (e.g., sodium channelopathies, GATORopathy‐spectrum disorders) could support cross‐syndrome or cross‐etiology stratification with potential utility for these designs.

**FIGURE 1:**
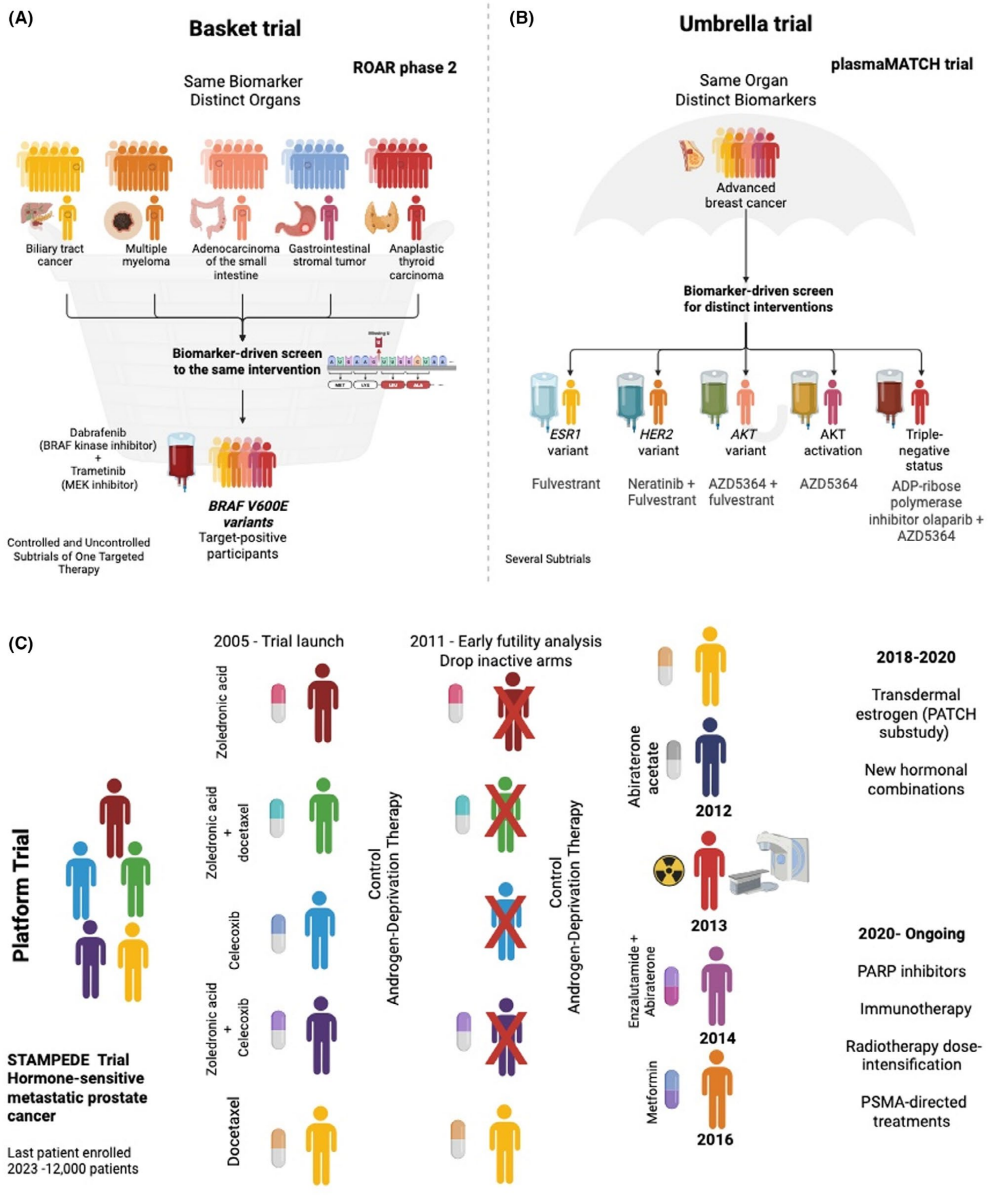
Principal subtypes of master protocol frameworks. Master protocols are designed to accelerate therapeutic evaluation using biomarker‐driven and adaptive methodologies. (A) *Basket trial*: ROAR, a single‐arm, histology‐agnostic phase II trial evaluating dabrafenib ± trametinib across multiple *BRAF V600E*–mutated tumor types. (B) *Umbrella trial*: plasmaMATCH, a molecularly stratified, single‐arm trial in advanced breast cancer assigning targeted therapies based on actionable genomic alterations (*ESR1, HER2, AKT, BRCA1/2*). (C) *Platform trial*: STAMPEDE, a perpetual multi‐arm, multi‐stage adaptive trial in prostate cancer allowed the continuous addition or discontinuation of treatment arms based on interim analyses. Together, these examples illustrate the continuum of adaptivity across master‐protocol subtypes, from fixed molecular baskets to stratified umbrella designs and fully adaptive platform trials. ADP, Adenosine diphosphate; MEK, Mitogen‐Activated Protein Kinase Kinase; PARP, Poly(ADP‐ribose) polymerase; PSMA, Prostate‐specific membrane antigen.

After a successful phase 2 randomized clinical trial,^8^ the US Food and Drug Administration (FDA) authorized a phase 3 multisyndrome trial for multiple DEEs.^9^ It marks a regulatory milestone that deserves recognition but also a critical analysis regarding its potential, limitations, and the evolving methodology of basket analog trials for DEEs, particularly for enabling phenotype‐based approaches to accelerate ethical and efficient treatment discovery.

## 1 CLARIFYING THE BASKET TRIAL DESIGN

Basket trials or bucket trials emerged in oncology as histology‐ or tumor‐agnostic trials evaluating the efficacy of a molecular or a biomarker‐driven targeted therapy across multiple cancer types, despite location. In single‐arm trials, all participants receive the same targeted therapy, and efficacy—typically measured by overall response rate or progression‐free survival—is evaluated within biomarker‐defined subgroups. In randomized trials, patients are assigned to the targeted therapy or a control arm, allowing direct comparison of efficacy outcomes and providing stronger evidence of treatment benefit.^10^

Therefore, baskets are not “mixed bags,”^11^ they represent rigorously designed studies conducted under a single master protocol, within which each basket functions as a predefined, hypothesis‐driven, and statistically independent substudy that may share information. The “basket” denotes a highly planned and systematic grouping based on shared biological rationale, not a random aggregation.^11^

## 2 TOWARD PATHWAY‐BASED PRECISION THERAPEUTICS IN NEUROLOGY

Basket trial methodology has been slowly and asymmetrically incorporated into neurology, with the earliest and most robust applications arising in neuro‐oncology. This trajectory mirrors that of medical oncology more broadly; tumors provide clear molecular targets, well‐established biomarkers, and quantifiable radiological endpoints, features that facilitate the operationalization of master protocols. For this reason, the field of neuro‐oncology currently offers the clearest and most mature examples of basket designs applicable to neurological disorders.

The multicenter abemaciclib basket trial represents one of the strongest demonstrations of a biomarker‐driven neurological basket protocol. Several central nervous system tumor groups harboring CDK4/6 pathway dysregulation were prospectively evaluated within a unified master protocol. The meningioma subgroup achieved 68.2% 6‐month progression‐free survival, markedly surpassing historical controls and prompting expansion of that cohort. In contrast, other subgroups—including gliomas and additional CDK4/6‐altered tumors—did not meet prespecified activity thresholds and were not advanced to expansion stages.^12^ This divergence illustrates the main principle of basket methodology; each subgroup constitutes an independent, hypothesis‐driven experiment designed to test a specific mechanistic prediction, rather than to guarantee uniform benefit across all biomarker‐linked diseases. This is precisely why the abemaciclib trial serves as the most instructive exemplar for neurology; it demonstrates the feasibility, the biological rationale, and the necessary selectivity that define successful basket architectures.

Beyond neuro‐oncology, early foundational work is emerging across other neurological domains.

In rare neurological diseases, a novel pharmacometrics‐informed trial simulation framework has been proposed to address settings where patient numbers are extremely limited and natural history data are sparse.^13^ Using autosomal recessive spastic ataxia of Charlevoix–Saguenay as a model, this approach integrates nonlinear mixed‐effects modeling and likelihood‐based testing to enhance statistical power and reduce bias in small, mechanistically similar cohorts. Such methodology could support multidisease basket trials across rare ataxias, leukodystrophies, and mitochondrial disorders, where mechanistic clustering is biologically plausible.^13^

In Alzheimer disease, trial eligibility increasingly mandates biomarker‐confirmed pathology such as amyloid positron emission tomography (PET) or tau PET imaging.^14^ This shift is effectively creating the conceptual foundation for future cross‐disease tauopathy trials, in which diverse neurodegenerative disorders linked by tau accumulation—progressive supranuclear palsy, corticobasal degeneration, frontotemporal lobar degeneration—could theoretically be evaluated under a single biomarker‐anchored therapeutic protocol.^14^

Collectively, these examples illustrate that although true basket trials are not yet widely implemented in neurology, the methodological tools, biomarker frameworks, and mechanistic rationales are gradually evolving. Neuro‐oncology provides the clearest proof of concept, and adjacent fields are now generating the biological and methodological prerequisites required to translate basket principles into broader neurological disease categories.

## 3 BEXICASERIN STUDY AS A TRANSITIONAL MODEL

Although the phase 3 bexicaserin trial (NCT06719141)^9^ has been mislabeled as a basket trial, it employs a cross‐syndromic inclusion strategy within a unified trial framework translating some of these methodological principles into epilepsy. The design enables broad generalizability while retaining regulatory robustness. Bexicaserin was granted the FDA Breakthrough Therapy Designation, underscoring unmet needs and regulatory receptivity.^9^

Unlike basket trials that cluster by molecular biomarkers, the DEE trial clusters by shared clinical phenotypes—severe, early onset, and drug‐resistant epilepsy with modification of neurodevelopment trajectory—representing network dysfunction rather than isolated etiologies or syndromes. These phenotypic anchors serve as unifying endpoints across heterogeneous DEEs.

The phase 3 bexicaserin trial (NCT06719141)^9^ is not a basket trial in the strict regulatory or methodological sense. Instead, it has a unified, cross‐syndrome phase 3 design that enrolls multiple DEEs under a common clinical phenotype: severe, early onset, pharmacoresistant epilepsy with developmental impairment. This structure retains classical phase 3 trial rigor (fixed intervention, randomization framework as applicable, prespecified endpoints) while incorporating features inspired by master protocols,^15^, ^16^ such as harmonized inclusion criteria across phenotypically similar but etiologically diverse DEEs. This design therefore represents a hybrid, cross‐syndromic phase 3 framework, bridging conventional syndrome‐specific trials and future mechanism‐ or phenotype‐anchored master protocols.

Although not a master protocol, it represents the evolving flexibility of regulatory agencies and trajectory of epilepsy trial methodology and provides a critical empirical anchor for the conceptual frameworks. This bold approach faces its challenges, as learnt from oncology.

## 4 LESSONS FROM ONCOLOGY: SUCCESSES AND FAILURES

### 4.1 Biomarker validity

As summarized in Table 1, the experience from oncology shows that basket trials succeed primarily when the biological premise is robust, the biomarker exerts a consistent functional effect across tissues, and the statistical and operational frameworks are sufficiently rigorous to detect true signals.^7^, ^10^, ^11^, ^12^, ^15^, ^16^ Conversely, failures tend to arise when the biomarker is not a true disease driver, when histology‐specific resistance mechanisms dominate, or when methodological limitations—such as underpowered variant‐specific cohorts or permissive inclusion of variants of uncertain significance—compromise interpretability. These examples illustrate how the strengths and weaknesses of basket trial methodology emerge directly from the interaction between (1) biological validity, (2) diagnostic precision, (3) statistical design, and (4) feasibility constraints. Such insights provide a conceptual foundation for considering how mechanism‐based trial structures might be adapted to genetically defined epilepsies, where similar issues of heterogeneity, developmental timing, and biomarker reliability will determine the feasibility of cross‐syndrome approaches.

**TABLE 1:** Reasons for success and failure of basket trials.^12^, ^13^, ^14^, ^15^

Dimension	Reasons for basket trial success	Representative examples	Reasons for basket trial failure/limitations	Representative examples
Biological rationale	Biomarker is a true driver with consistent functional impact across histologies	*NTRK* fusions → larotrectinib with high ORR across >25 tumors	Biomarker not a driver, or effect is highly context‐dependent	*PIK3CA* heterogeneous mutations; weak drivers
Histological independence	Drug response largely tissue‐agnostic; histology does not modify efficacy	*BRAF* V600E inhibition in histiocytoses, NSCLC	Histology‐specific resistance overrides biomarker–drug interaction	*KRAS* G12C: reduced efficacy in CRC due to EGFR feedback
Variant‐level predictive value	High pathogenicity; variant subclass highly predictive	Class I *BRAF* alterations	Presence of VUS, mixed allelic functionality, or coalterations diluting signal	SUMMIT trial: variable responses to HER2 inhibitors
Quality of molecular profiling	Robust DNA/RNA integration; reliable detection of fusions and rare variants	RNA‐based *NTRK*/*NRG1* fusion detection	Incomplete profiling, missing complex fusions, or false positives (e.g., clonal hematopoiesis)	cfDNA false positives
Study design	Adaptive multicohort structures; preplanned statistical decision rules	MyPathway; TAPUR	Underpowered cohorts; inconsistent eligibility; unstable pooling strategies	Multiple MATCH arms closing for low accrual
Statistical methodology	Bayesian hierarchical models allow information‐borrowing while controlling false discovery; multiplicity well managed	Hierarchical modeling in early phase pan‐cancer designs	Inadequate control of type I error, post hoc “cherry‐picking,” simplistic pooling	Early basket trials without error rate control
Resistance biology	Predictable, potentially targetable resistance mechanisms	TRK inhibitors with MAPK escape manageable by combination therapy	Strong tissue‐specific or pathway‐level escape mechanisms	EGFR‐mediated resistance in *BRAF*‐mutant CRC
Operational feasibility	Broad multicenter enrollment; efficient molecular‐to‐trial matching; inclusion of pediatric/rare tumors	Global larotrectinib program enabling rapid accrual	Limited site activation; geographic disparities; slow recruitment for ultrarare variants	FGFR inhibitor trials outside cholangiocarcinoma
Generalizability	Molecular mechanism broadly conserved; population is representative of real‐world biomarker carriers	MSI‐H/MMRd approvals	Highly selected populations (narrow eligibility criteria) limit external validity	Ultrarare tumors with idiosyncratic biology
Clinical & molecular heterogeneity	Subsets share convergent biology that reduces variability	NTRK‐fusion tumors with similar TRK‐dependence	Heterogeneity in tumor microenvironment, comutations, or natural history undermines pooling	Differences in HER2‐driven tumors across organs
Regulatory considerations	Clear tissue‐agnostic rationale; reproducible effect across cohorts supports approval	FDA approvals for NTRK, MSI‐H	Regulatory uncertainty; inconsistent evidentiary standards across biomarkers	Ambiguous results for TMB‐high tumors
Site‐level logistic	Trials integrated into specialized molecular networks; streamlined diagnostics	National NGS‐driven matching platforms	Difficulty embedding basket trials into organ‐specific clinical workflows	Traditional oncology clinics organized by tumor type
Evolving standard of care	Stable comparator landscape simplifies interpretation	Early TRK inhibitor development	Changing SoC complicates interpretation and temporal consistency	Immunotherapy‐era shifts in MSI‐H CRC management
Missing data/practical barriers	High trial coordination, strong data completeness	Centralized molecular profiling hubs	Missing data, incomplete phenotyping, variable imaging/response criteria	Multisite rare‐variant basket trials
Sample size constraints	Large enough cohorts in common variants; hierarchical borrowing supplements rare subsets	*BRAF* V600E basket across multiple cancers	Very small rare‐variant cohorts yield unstable estimates; limited inference	Ultrarare fusions with *n* < 5 per arm

*Note*: Operational feasibility, site‐level logistics, and missing data/practical barriers are categories of operational and practical feasibility. These dimensions represent implementation‐level determinants of basket trial performance rather than biological or statistical factors.

Abbreviations: BRAF, v‐raf murine sarcoma viral oncogene homolog B1; cfDNA, cell‐free DNA; CRC, colorectal cancer; EGFR, epidermal growth factor receptor; FDA, US Food and Drug Administration; FGFR, fibroblast growth factor receptor; HER2, human epidermal growth factor receptor 2; *KRAS* G12C inhibitors, the *KRAS* gene trial showed reduced efficacy in colorectal cancer due to reactivation of epidermal growth factor reactivation; MAPK, mitogen‐activated protein kinase; MATCH, Molecular Analysis for Therapy Choice; MMRd, mismatch repair deficiency; MSI‐H, microsatellite instability–high; NGS, next generation sequencing; NRG1, neuregulin 1; NSCLC, non‐small‐cell lung cancer; NTRK, neurotrophic tyrosine receptor kinase; ORR, objective response rate; PIK3CA, phosphatidylinositol‐4,5‐bisphosphate 3‐kinase catalytic subunit alpha; SoC, standard of care; TAPUR, Targeted Agent and Profiling Utilization Registry; TMB, tumor mutational burden; TRK, tropomyosin receptor kinase; VUS, variant of uncertain significance.

### 4.2 Study design and classical models

From a methodological perspective, basket trials can be understood as quasiexperimental designs in which genetically defined subgroups share a common mechanism of disease and receive the same intervention. The analytical challenge is therefore the comparison of multiple groups under a unified biological framework, a problem that classical statistics has addressed for decades.^7^, ^10^, ^11^, ^12^, ^15^, ^16^ For numerical outcomes, models such as analysis of variance with appropriate post hoc procedures (Tukey for homoscedasticity, Games–Howell for heteroscedasticity, or Steel–Dwass variants for nonnormal data) remain robust and transparent. For categorical outcomes, logistic or multinomial regression with modern multiplicity adjustments—preferably Holm rather than overly conservative Bonferroni corrections—achieves adequate control of type I error. These methods do not eliminate false‐positive risk but use distributions that explicitly incorporate the number of comparisons, thereby limiting the inflation that arises when multiple hypotheses are evaluated simultaneously.^7^, ^10^, ^11^, ^12^, ^15^, ^16^, ^17^

### 4.3 Frequentist versus Bayesian approaches and hierarchical models

Frequentist and Bayesian frameworks are applicable to basket trials.^17^ Frequentist inference treats population parameters as fixed and unknown, providing point estimates, confidence intervals, and *p*‐values derived entirely from the likelihood. Bayesian inference incorporates prior knowledge through probability distributions, producing posterior estimates and credible intervals, but depends heavily on the justification and specification of these priors, an issue that becomes critical when sample sizes are small. Contrary to common belief, Bayesian models do not “solve” the small‐sample problem. Bayesian methods reweight information toward the prior when data are sparse or weak, which can stabilize inference but does not necessarily increase precision. The resulting estimates depend strongly on how well the prior reflects the underlying biological or statistical context.

In settings where genetically defined subgroups may share correlated responses, hierarchical or multilevel models—estimated either through mixed‐effects frequentist methods or Bayesian hierarchical structures—allow partial pooling of information while acknowledging subgroup heterogeneity. Such models rely on the assumption of exchangeability among subgroups, which must be explicitly justified in light of biological plausibility and prior evidence. These approaches do not replace careful design or adequate sample size but provide coherent ways to model the biological relationships that motivate basket trials in the first place.

## 5 REGULATORY DIVERGENCIES

The FDA and the European Medicines Agency (EMA) have recognized the potential of master protocols for the development of innovative cancer therapies; however, their regulatory frameworks diverge significantly.

The FDA's Complex Innovative Trial Design initiative is part of its broader strategy to enhance the efficiency and speed of the drug approval process. The FDA aims to modernize protocols allowing for flexibility in evidence generation. This approach supports the integration of biomarker‐driven patient selection, providing an opportunity for more personalized treatments to enter the market more quickly.^18^

Additionally, the FDA's Real‐Time Oncology Review (RTOR) accelerates the review process, allowing clinical data assessment as it becomes available, rather than waiting for the completion. The success of RTOR is a paradigm shift in an era when timely patients' access to innovative cancer treatments is critical.^19^

The EMA, through its Adaptive Pathways and Priority Medicines (PRIME) program, allows for a stepwise licensing process, enabling earlier access to new drugs by granting conditional approvals based on preliminary data, while requiring ongoing studies to confirm benefit–risk balances postapproval. The PRIME program was launched to prioritize and provide guidance for drugs addressing unmet medical needs, underscoring the commitment to facilitating innovative therapeutic solutions while ensuring rigorous safety and efficacy evaluations. Evidence generated through studies within the PRIME framework is anticipated to be adapted based on real‐world data, aligning closely with patient needs and outcomes.^20^

In summary, both the FDA and EMA are modernizing the drug approval landscape with their respective initiatives, although with distinctive methodologies and regulatory frameworks. Whereas the FDA focuses on accelerated timelines and flexible designs, the EMA emphasizes iterative evidence generation alongside stringent postmarketing obligations. This regulatory divergence holds particular importance for neurology; as basket trial frameworks evolve, parallel dialogue with agencies will be critical to harmonize endpoints and extrapolation strategies.

A critical and underdiscussed regulatory divergence with direct implications for epilepsy drug development concerns the treatment of adult and pediatric populations as separate licensing pathways. Whereas oncology increasingly permits age‐spanning, tissue‐agnostic approvals when disease biology and treatment response are conserved, neurology remains largely bound to sequential adult‐first authorization, followed by delayed pediatric evaluation. Both the FDA and EMA acknowledge the limitations of this approach for early onset, lifelong disorders such as DEEs and, consistent with the principles formalized in the ICH E11A Pediatric Extrapolation Guideline (2022)^21^ and the EMA's recent extrapolation frameworks,^22^, ^23^ have adopted structured approaches to support pediatric extrapolation when scientific similarity across ages is sufficiently strong. In the FDA model, the extent of extrapolation ranges from full (requiring only pharmacokinetic and safety data) to partial (requiring limited pediatric efficacy data), depending on similarity in disease progression, pharmacology, and exposure–response relationships. The EMA applies the same scientific principles within the regulatory architecture of the Pediatric Investigation Plan, which operationalizes extrapolation strategies through model‐informed pharmacokinetic–pharmacodynamic bridging, ontogeny‐aware physiological modeling, natural history data, and iterative evidence generation. When biological continuity across development is well supported, harmonized age‐range licensing becomes feasible, as illustrated by the recent bexicaserin authorization from 2 to 65 years.^9^ For master protocol approaches in epilepsy—whether mechanism‐anchored or phenotype‐defined—harmonized regulatory strategies are essential to avoid duplicative trials, ensure timely access for children, and align approval pathways with the inherent biological unity of early life DEEs.

## 6 FROM MECHANISM TO PHENOTYPE‐DEFINED BASKETS: CONCEPT AND IMPLEMENTATION

The basket conceptual framework is relevant to epilepsy, particularly to monogenic epilepsies, where heterogeneous clinical phenotypes may arise from convergent molecular mechanisms.^24^ In this context, the use of the basket concept is metaphorical; phenotype‐anchored "baskets" are conceptually aligned with biomarker‐defined designs but remain methodologically distinct, as their unit of stratification is clinical phenotype rather than molecular driver. Similar to oncology, success in neurology will depend on identifying alterations that function as true “disease drivers” rather than secondary or modifying influences.^25^ Molecularly defined groups may form the closest neurological analogue to “baskets.”

However, the translation is not straightforward. Unlike cancer, where a single dominant driver variant controls tumor growth, genetic epilepsies involve complex neurodevelopmental trajectories shaped by function (gain vs. loss), timing, cellular context, and coexisting genetic modifiers.^26^ This increases the risk that a shared biomarker might not predict uniform drug response across diverse phenotypes, age groups, or developmental stages. The challenges observed in oncology—coalterations and context‐dependent pathogenicity—appear in epilepsy as variable expression patterns, network reorganization, differences between cortical and subcortical circuits, and age‐dependent phenotypic expression.^26^ These factors can undermine the tissue‐agnostic assumption and must be addressed when designing mechanism‐based neurological trials.

Statistical considerations also map directly onto epilepsy. As in oncology, randomization becomes difficult when standards of care vary across epilepsies, ages, or countries.^10^, ^15^, ^16^ Most precision‐medicine trials in epilepsy will be early phase, small, and nonrandomized, risking underpowered subgroup analyses and overinterpretation of heterogeneous responses.^10^, ^15^, ^16^ Bayesian hierarchical models—already used in rare‐disease neurology—may support “borrowing of information” across genetically related cohorts (e.g., across sodium channel disorders), while maintaining control of false discoveries.^17^ Adaptive multistage designs are likely essential, allowing discontinuation of inactive genetic cohorts and concentrating statistical power on the most biologically plausible responders.^10^, ^15^, ^16^

Finally, the feasibility challenges are even more pronounced in epilepsy than in oncology. Rare variants, early childhood onset, developmental comorbidities, and the need for longitudinal electroencephalography‐based outcomes complicate recruitment and retention. Harmonization of outcome measures, careful phenotypic stratification, and precise biomarker definitions (e.g., genotype–phenotype correlations, electrophysiologic endophenotypes) are necessary to avoid the pitfalls that have limited basket trials in oncology.

In summary, basket trial principles are highly applicable to epilepsy, particularly in monogenic DEEs, but require greater attention to factors associated with phenotypic heterogeneity. If these factors are incorporated into design, mechanism‐based cross‐syndrome therapeutic studies may become a viable route for precision medicine in rare epilepsies.

## AUTHOR CONTRIBUTIONS

Kette D. Valente is the sole author and is responsible for the conception of the study; the acquisition, analysis, or interpretation of data for the work; drafting the work or revising it critically for important intellectual content; final approval of the version to be published; and ensuring that questions related to the accuracy or integrity follow International Committee of Medical Journal Editors guidelines.

## FUNDING INFORMATION

K.D.V. is the recipient of a grant from Fundação de Amparo à Pesquisa do Estado de São Paulo (2021‐14144‐0).

## CONFLICT OF INTEREST STATEMENT

K.D.V. has been paid as a consultant/speaker or has received support from Biocodex, Takeda, UCB, Praxis, Lundbeck‐Longboard, and Latin American Pharmaceutic Companies. She has been the principal investigator for trials by Prati‐Donaduzzi, Takeda, Praxis, and Lundbeck‐Longboard. These activities are not related to this article. I confirm that I have read the Journal's position on issues involved in ethical publication and affirm that this report is consistent with those guidelines.
